# First lactation performance of sows in Maternity Ring housing is comparable to that in farrowing crates

**DOI:** 10.3389/fvets.2025.1717512

**Published:** 2026-02-04

**Authors:** Kate J. Plush, Kirsty L. Chidgey, Nigel Young, Darryl N. D’Souza, Robert J. van Barneveld

**Affiliations:** 1 SunPork Group, Eagle Farm, QLD, Australia; 2 School of Agriculture and Environment, Massey University, Palmerston North, New Zealand

**Keywords:** sow, welfare, farrowing, performance, pen, piglet survival, maternity ring

## Abstract

There is a trade-off between sow and piglet welfare in farrowing pens, whereby increased behavioural freedom of sows results in unacceptable levels of piglet mortality. Improvements in sow welfare have been demonstrated in such housing systems; however, the footprint is often at least 50% greater than a standard farrowing crate. This remains an impediment to the adoption of confinement-free farrowing. The Maternity Ring, a system with a similar footprint to a farrowing crate, was designed to avoid close confinement of sows. However, its performance, particularly with regard to piglet survival, has not yet been quantified but is an essential part of evaluating commercial viability and acceptability in terms of piglet welfare. This experiment’s aim was to determine whether piglet mortality differed between farrowing crates and Maternity Rings. First-parity sows were recruited over 12 months and randomly allocated to one of the two treatments: farrowing crate (FC; *n* = 184) and Maternity Ring (MR; *n* = 205). Litter size and piglet mortality (number, age, and cause of death), as well as piglet fostering movements and medical interventions for sows and litters, were recorded. There was no difference in total pigs born, pigs born alive, or the number of pigs weaned between the two treatments. There was a tendency for a 0.3-pig-per-litter increase in pre-foster mortality in MR sows (*p* = 0.065), but pigs born dead, post-foster deaths, liveborn mortality, and total deaths were similar to FC sows. Piglet removal for ill thrift was 0.3 pigs per litter lower in MR sows (*p* = 0.05), and the incidence of medications in litters was reduced from 62 to 50% (*p* < 0.05). Additionally, sows were medicated less frequently in MR (6%) than in FC (15%, *p* < 0.05). MR housing achieved comparable liveborn piglet mortality to FC in first-parity sows. Future studies should test whether this performance is repeatable as sows are managed across multiple parities.

## 1 Introduction

Farrowing crates are a housing system used in the pork industry to accommodate sows and piglets during parturition and lactation. Farrowing crates were adopted to facilitate efficient sow and litter management and care, improve piglet survival, and make efficient use of building space, which is a significant cost when establishing an indoor pork production unit. A key objective of the farrowing crate is to closely confine the sow to reduce the frequency and speed of posture changes, thereby reducing piglet deaths attributed to accidental crushing (1). Preventing crushing via farrowing system design has been a priority since crushing is the most common cause of liveborn piglet mortality and is associated with significant welfare compromise. A large study based on records from 2,143 litters reported that the crushing of healthy piglets was the most common cause of liveborn mortality (54.8%), followed by low viability (13.8%), starvation (6.8%), and unknown causes (6.1%) (2). Following necropsy, the pathological findings of piglets that died due to crushing included thoracic and/or abdominal internal bleeding, bruising, and skull and rib fractures (3). The likely welfare impacts associated with these findings include negative experiences such as pain, fear, panic, breathlessness, and helplessness. Consequently, both the severity of the associated welfare impacts and the frequency of crushing as a cause of death of piglets have cemented piglet crushing as a significant economic and welfare challenge.

Nonetheless, confining sows within a farrowing crate has negative welfare implications, evidenced by severe limitations in physical movement and normal behaviour. The resulting welfare compromises for sows include pain and discomfort from shoulder sores, locomotor problems, prolonged farrowing duration, and frustration due to inhibited natural behaviours such as nest building, maternal behaviour, and piglet contact (4). Increasing the sow’s freedom of movement during farrowing and lactation by introducing an alternative lactation housing system, such as a farrowing pen, is often associated with an increase in piglet mortality. In a meta-analysis by Glencorse et al. (5), the relative risk of piglet mortality was 14% higher in farrowing pens than in farrowing crates.

We have previously argued that opportunities exist to improve piglet survival in farrowing pens through several key design aspects, namely anti-crushing bars or railings that provide escape zones for piglets during sow posture changes, clearly defined piglet and sow zonation for thermal comfort, and the correct amount of dedicated pen space to achieve these zones as early in lactation as possible (6). Baxter et al. (7) compared piglet survival in pens with differing nest area sizes: either 3.3m^2^ (small) or 4.0m^2^ (large). The space provided influenced liveborn pre-weaning piglet mortality, which was significantly greater in the larger nest area (18.1%) compared to the smaller nest area (10.9%). Nest size influences the overall footprint of the farrowing space, and therefore, larger nest areas that provide more opportunities for sow movement and behavioural interactions may delay the piglets from locating the creep, prolonging their exposure to an area that does not meet their thermal needs. Despite the availability many commercially available farrowing pens, challenges remain in meeting all three key aspects: improving sow welfare, supporting piglets to locate the creep area quickly, ensuring that creep usage by piglets is high from the moment of birth to keep them out of the sow zone, and when piglets are in the sow zone, providing escape areas that they can use when the sow changes posture.

The Maternity Ring is a close confinement free farrowing system that has demonstrated advantages in sow welfare compared to the farrowing crate, as measured objectively using the Five Domains framework (8). The Five Domains model of animal welfare assessment considers the physical environment, nutrition, health/ functional status, and behavioural interactions, with the resulting experiences in these first four domains culminating in the fifth domain to describe welfare status through emotional state or affective experiences (9). Sows housed in a Maternity Ring had more positive affective experiences than those in a farrowing crate (8). To summarise, these experiences included a greater propensity to observe and interact with the environment both within and outside the pen, the ability to perform species-specific behaviours such as nesting and piglet bonding, and a reduced defence cascade response. In addition to clear animal welfare benefits, the design of the Maternity Ring incorporated the aspects outlined above that could potentially limit the amount of piglet mortality that is traditionally reported in other free-farrowing systems.

This experiment aimed to compare the performance of first-parity sows housed in a Maternity Ring to those in a farrowing crate, with a specific focus on the timing and causes of piglet mortality. We hypothesised that sows housed within a Maternity Ring would achieve similar piglet survival rates to those in a farrowing crate.

## 2 Materials and methods

### 2.1 Animals and management

The experiment was conducted between January and December 2024 on a 1,000-sow unit (SunPork Farms, Darfield, NZ) that operated a two-weekly batch farrowing system of 110 sows per batch. Two sides of the farrowing house contained five identical, mechanically ventilated rooms, with each room holding 22 farrowing spaces with 18 h of artificial light and 6 h of darkness daily. Each farrowing space housed a single sow and her litter, was installed over metal slatted flooring, and contained a piglet creep with solid plastic mat flooring, one piglet drinker, two sow drinkers, and one sow feeder. Sows were moved into the farrowing house approximately 5 days before their expected due date. Sows in both treatments were provided a hessian sack (0.45 × 0.73 m in size) as manipulable material for nesting. This hessian was affixed to a middle bar close to the feeder in the farrowing crate and to the ring in the corner opposite the feeder in the Maternity Ring. Piglets were weaned at 22.3 ± 1.72 lactation days. Prior to and for the first 3 days after farrowing, sows were fed a transition diet (13.6 MJ DE/Kg, 0.0547 SID Lys/DE %/MJ) twice daily in a “step up” regime. A lactation diet (14 MJ DE/Kg, 0.062 SID Lys/DE %/MJ) was introduced at 4 days post-farrowing using the same regimen until *ad libitum* intakes were achieved 6 days after farrowing. Piglets were provided with creep feed in bowls from approximately 10 days of age. Sows farrowed naturally (no hormonal induction or assistance) and were fostered to rearing capacity approximately 24 h after farrowing completion. Minimal fostering was performed, and piglets were only moved to ensure access to a functional teat. Piglets were tail docked and given an intramuscular iron injection at 3 days of age.

### 2.2 Experimental design and housing

**Figure 1:**
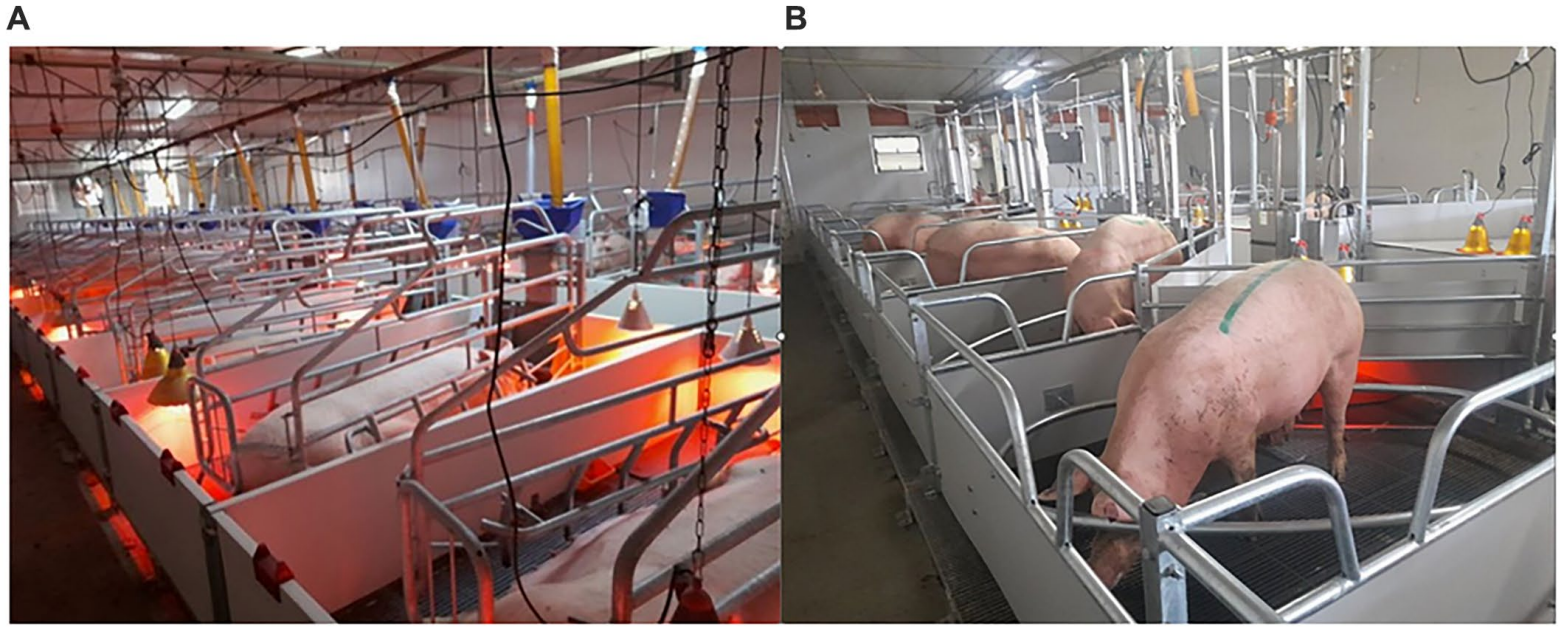
Two identical farrowing rooms utilised by experimental sows containing **(a)** farrowing crates or **(b)** Maternity Rings.

Only first-parity sows (Camborough 29; PIC, Darfield NZ) were recruited into the experiment. Subsequent investigations will follow the same experimental sows through the two treatments to later parities. Two rooms in each of the two-weekly batches were recruited for the experiment (Figure 1). One of these rooms contained 22 farrowing crates (1.45 m × 2.62 m) with a sow stall (0.6 m × 1.8 m) on a 45-degree angle to facilitate front corner piglet creep (FC, *n* = 184 sows). The other room contained 18 Maternity Rings (1.8 m × 2.4 m, Stockyard Industries, North Bendigo, VIC, AU) with the design incorporating an oval ring (2.06 m × 1.16 m) fixed at a height of 0.25 m above the floor, which allowed the sow to turn around and protected piglets from crushing (MR; *n* = 205 sows).

### 2.3 Measurements

Production data were collected for the number of piglets contributed (total pigs born, number born alive, and number born dead) and the number of piglets weaned per sow. The number of pigs lost was also recorded (pre-foster deaths, post-foster deaths, liveborn deaths, and total deaths per litter). Causes of death were categorised as savaged, low viability, crushed, chilled, starved, deformed, illness, and “other” (2) and were evaluated and recorded for each piglet by trained stockpersons. If a piglet began losing body condition, it was removed, categorised as “ill thrift,” and placed on a nurse sow outside of the experiment. Health and welfare monitoring was conducted twice daily after feeding. A sow or piglet displaying symptoms of illness—sow: fever, vaginal discharge, mastitis, or lameness; piglet: scour, meningitis, ill thrift, or physical injury—was medicated using the farm’s approved veterinary medication list, which generally was an antibiotic and non-steroidal anti-inflammatory drug, and these were recorded.

### 2.4 Statistics

All data were analysed in SPSS v29 (IBM; Armonk, NY, United States) with a *p*-value of < 0.05 deemed significant, and a *p*-value of < 0.10 was the trend. Count data included total piglets born, piglets born alive, piglets born dead, number of piglets weaned, fostering events (at birth and removal for ill thrift) as well as time of mortality (born dead, pre-foster deaths, post-foster deaths, liveborn deaths, and total deaths), and cause of mortality (savaged, low viable, crushed, chilled, starved, deformed, illness and other). The data were analysed using negative binomial regression. Incidence data, including sows and piglets medicated, were analysed using binary logistic regression. The model applied was the same for all traits and included the farrowing season (summer, autumn, winter, and spring) and treatment (FC and MR).

## 3 Results

**Figure 2:**
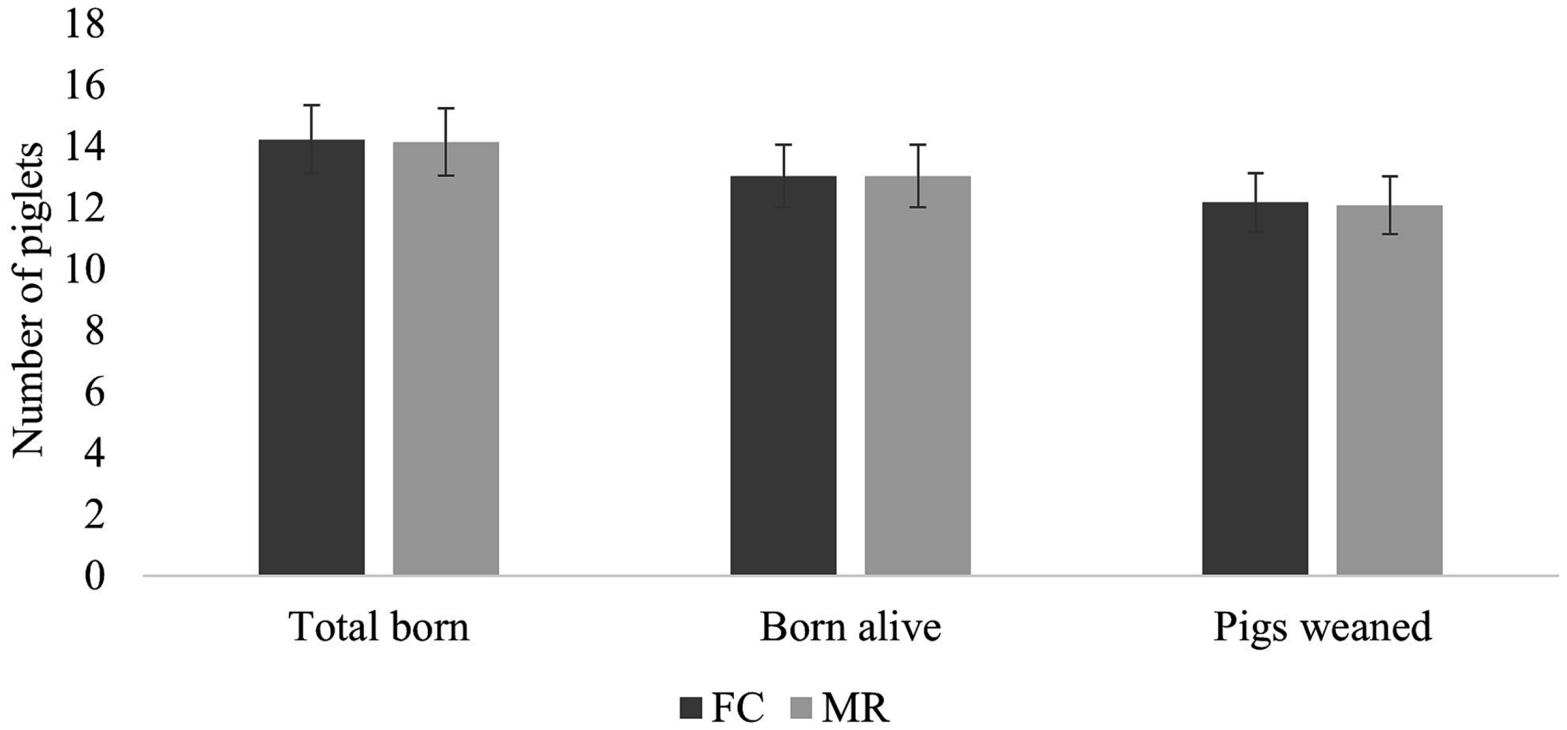
Number of piglets contributed (total born, born alive, and weaned) per litter (mean ± SEM per sow) for both farrowing crate (FC) and maternity ring (MR) treatments.

There was no significant difference in piglets contributed (total born or born alive) between FC and MR sows (Figure 2). Similarly, there was no significant difference in the number of pigs weaned between the two treatments.

**Figure 3:**
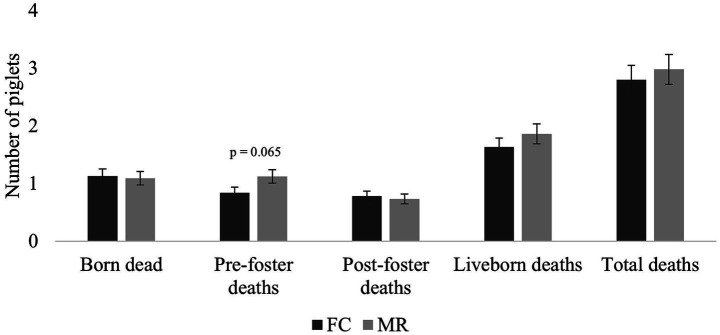
Piglet mortality (mean ± SEM per sow) during birth (born dead), prior to any piglet movement (pre-foster deaths), after the movement of piglets for litter equalisation (post-foster deaths), liveborn (the sum of pre-foster and post-foster deaths), and total (sum of born dead and liveborn deaths) for farrowing crate (FC) and maternity ring (MR) treatments.

The number of piglets born dead per litter was similar in both treatments (Figure 3). There was a tendency (*p* = 0.065) for fewer piglet deaths before fostering in the FC sows (0.8 ± 0.10) than MR sows (1.1 ± 0.12). After fostering, once more, there was no effect of treatment on piglet mortality. Total piglet losses per sow did not differ between FC and MR sows.

Stockperson determination of the cause of piglet deaths is outlined in Table 1. The only treatment effect observed was the incidence of crushing, with 0.6 more pigs per litter crushed in MR sows than in FC sows (*p* < 0.001).

**Table 1:** Cause of piglet death (mean ± SEM per sow) as determined by stockpeople for farrowing crate (FC) and maternity ring (MR) treatments.

Cause	FC		MR		
	Mean	SEM	Mean	SEM	*P*-value
Savaged	0.1	0.03	0.0	0.00	0.17
Low viability	0.4	0.06	0.4	0.05	0.55
Crushed	0.3	0.06	0.9	0.10	< 0.001
Chilled	Incidence too low				
Starved	0.0	0.01	0.0	0.01	0.27
Deformed/injury	0.2	0.04	0.2	0.03	0.87
Illness	0.1	0.04	0.1	0.03	0.46
Other	0.1	0.03	0.0	0.01	0.65

**Figure 4:**
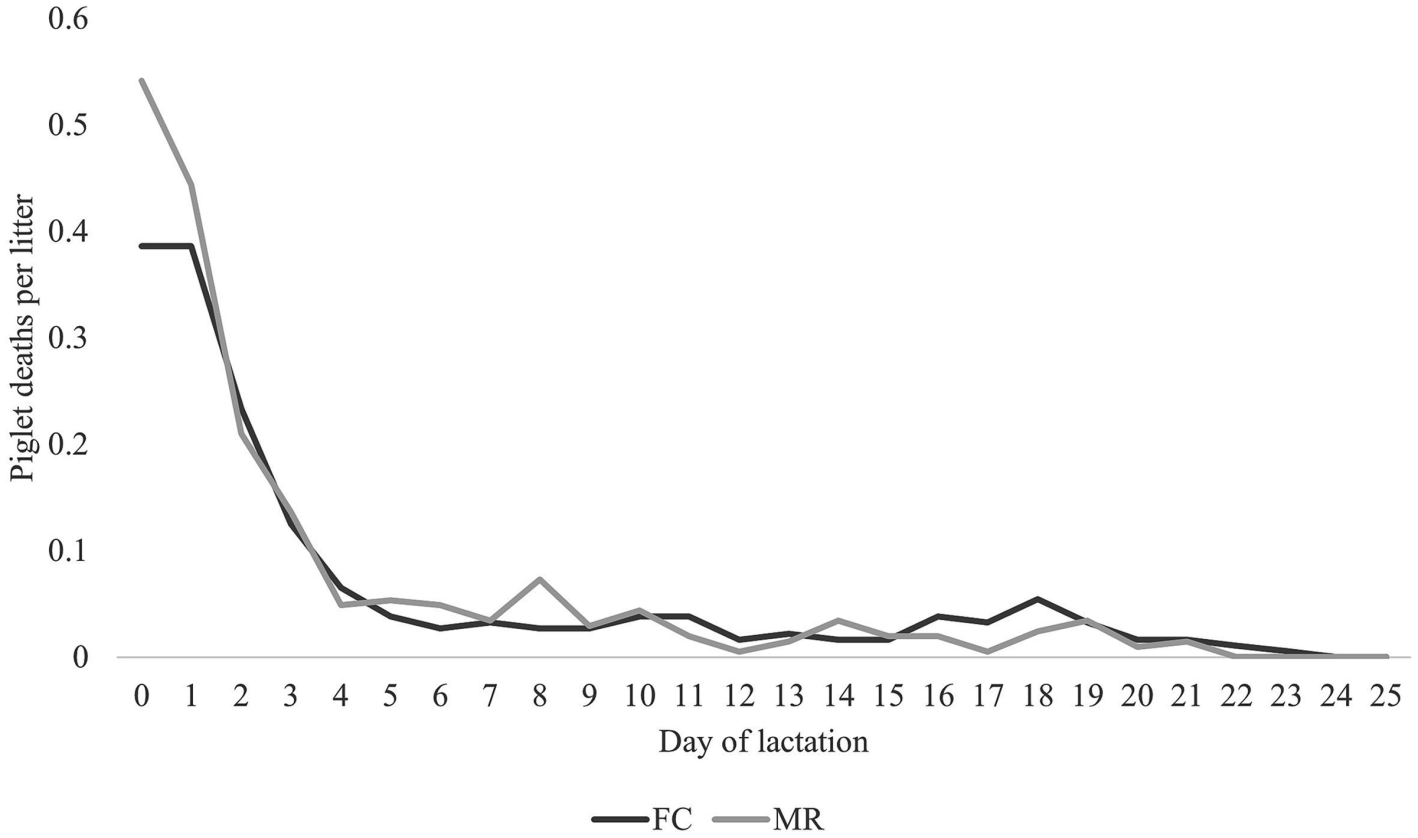
Number of piglet deaths (mean ± SEM per sow) during each day of lactation for farrowing crate (FC) and maternity ring (MR) treatments.

Piglet losses were 0.2 pigs per litter greater on the day of farrowing (day 0) and 0.1 pigs per litter greater on day 1 in MR than in FC (*p* = 0.065). However, no differences in piglet mortality could be established after this initial stage of lactation (Figure 4).

**Figure 5:**
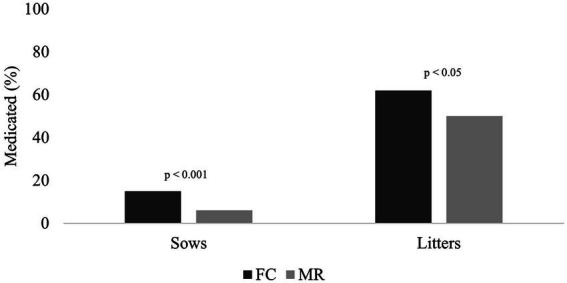
Mean percentage of sows and litters medicated during lactation for farrowing crate (FC) and maternity ring (MR) treatments.

The incidence of sows medicated during lactation was 15% (95% confidence interval 10–22%) for FC and 6% (95% confidence interval 4–11%) for MR treatments (Figure 5; *p* < 0.01). The incidence of litter medications was higher in FC sows than in MR sows (*p* < 0.05, with 62% (95% confidence interval 54 70%) for FC litters and 50% (95% confidence interval 43–58%) for MR litters.

There was a tendency to foster fewer piglets in MR (0.2 ± 0.09 pigs per litter) than in FC (0.4 ± 0.09 pigs per litter, *p* = 0.053). A significant difference in piglets fostered off due to ill thrift was identified, which was greater in FC (0.7 ± 0.12) than in MR (0.4 ± 0.12) litters (*p* = 0.05).

## 4 Discussion

We hypothesised that MR sows, housed within the same footprint as an FC but without close confinement, would achieve similar piglet survival rates as crated sows. Our results show no difference in the number of pigs born and born alive between treatments. Although there was a tendency for higher piglet losses in the first days after farrowing in MR than in FC, this did not translate into differences in overall liveborn mortality, and the number of pigs weaned was maintained. This study therefore supports the overarching hypothesis that piglet survival—and consequently animal welfare standards as well as production output—can be maintained in a close-confinement free system, such as the MR, even when it occupies the same space allowance as an FC.

There has been a strong scientific focus on the positive relationship between space allowance and piglet survival, which originally stemmed from the idea that sows need adequate space to “group” piglets prior to lying down to prevent crushing deaths (10). In an analysis of more than 12,000 litters across almost 10 farms, Weber et al. (11) showed that pen size was not a significant factor for piglet mortality or mortality due to crushing and concluded that piglet losses were more closely related to characteristics of the sow than the design of the farrowing pen. Similarly, Glencorse et al. (5) identified no significant impact of pen size on piglet survival in their meta-analyses conducted across experiments examining the impact of farrowing accommodation on piglet survival. In our recent review, when compared within the same experiment, increased nest area was negatively related to pre-weaning mortality (6). This was best demonstrated by Baxter et al. (7), who showed that by increasing the length of the nest area from 2.3 m to 2.8 m, pre-weaning piglet mortality was increased by approximately 8%. A “slight trend” in decreasing mortality was identified by EFSA (12) only when pens with less than 4 m^2^ were discounted from the analyses. These authors also modelled that making 4 m^2^ available to the sow would result in a pre-weaning mortality of 20%, whilst 6.6 m^2^ would result in similar performance to a crate. Our results are in clear opposition to this modelling, with the MR providing the sow 3.83 m^2^ and achieving a piglet mortality of 13.8%, comparable to the FC, which achieved a mortality of 13.5% (calculated as all liveborn piglet deaths divided by all pigs born alive × 100).

The stillbirth rate was on average 7.7% in the present study, which is within the expected range of 5–10% (13). It was not possible to determine the proportion of type I (prepartum) versus type II (intrapartum) stillborn deaths, and whether these differed between treatments, as no detailed necropsy was conducted. It is also of note that only first-parity sows were used in our study. A comparison between sows in farrowing crates and those in free-farrowing pens reported a similar trend of comparable piglets born, including total born and born alive (14). A similar trend was observed in another study reporting the performance of sows in farrowing crates and in pens with temporary confinement for 3 days post-farrowing (15). Both experiments compared different systems on the same farm, as was the case with our study. Others have reported a beneficial effect of loose farrowing on the stillbirth rate, such as Nowland et al. (16), whereby the number of stillborn piglets per litter was halved in unconfined (0.2 piglets/litter) versus confined sows (0.4 piglets/litter). Given that the incidence of stillborn piglets increases with parity (13), our ongoing study will document the performance of this herd with older sows. This will help detect whether the lack of a treatment effect on stillborn piglets is real or whether there is any consequence of repeated exposure to a particular farrowing system.

There was a tendency for fewer piglet deaths before fostering in the FC sows compared to the MR sows. Moustsen et al. (17) and Hales et al. (18) also found that liveborn piglet deaths were greater before litter equalisation in loose sows compared to sows that were confined during parturition. Furthermore, the number of piglets that survived to day 10 was significantly lower in loose sows versus those either confined temporarily or permanently (17). In the present study, piglet survival did not differ after the initial fostering event. As such, the results of our study are contrary to most published literature, where piglet survival at weaning is reportedly lower in free-farrowing systems versus pens with temporary or permanent crating.

When the causes of piglet death were examined, it was apparent that 0.6 more piglets per litter were crushed in MR than in FC. This was the only significant treatment effect across all causes of death recorded. This finding was not unexpected, given that many existing publications have reported increased piglet deaths due to crushing in free farrowing versus crated systems, largely due to greater sow mobility (14, 19–21). Once again, this effect was only observed during early lactation in the present study, and after fostering, there was no difference between systems for the number of piglets that died due to crushing. This is unlikely to be a direct effect of fostering; rather, fostering at 24 h of age coincides with a drop in pre-weaning mortality across all crated, free-farrowing, and outdoor systems (2). We suspect that this tendency for increased piglet crushing in the early stages of lactation is due to two main factors, both attributed to the experimental cohort being primiparous animals. First, primiparous sows are more reactive to piglets, with this effect being stronger in a free-farrowing environment (22), which may increase the risk of crushing by changes in sow behaviour. Second, piglets born to first-parity sows are lighter and fail to ingest as much colostrum as those born to multiparous sows (23), again putting them at risk of crushing by increasing the likelihood of being within proximity to the sow. Future studies will confirm or question the finding of increased crushing in early lactation, as this sow cohort will be followed through the same two housing systems for subsequent parities.

The administration of medications to piglets was 12% lower in MR than in FC. This finding is similar to that of our earlier work (8) and was attributed to a lower incidence of meningitis (*Streptococcus suis*) in MR piglets. The primary reason for medicating piglets in the present study in both systems was for the treatment of diarrhoea (likely *Escherichia coli*). First-parity sows produce less colostrum (24) with a reduced quantity of immunoglobulins, which has been associated with diarrhoea in piglets (25). Interestingly, Nowland et al. (16) reported that piglets born in a free-farrowing system ingested more colostrum than piglets reared in farrowing crates. This finding may explain why fewer MR piglets were medicated compared to FC piglets. An alternative explanation for a reduced incidence of medications could be the stockperson’s behaviour. Staff safety concerns arise when potentially defensive behaviour of sows is directed towards people, with the risk to the stockperson being higher when the crate is removed in free-farrowing systems. A hesitation to enter the MR may inhibit the spread of disease, which is positive, but on a negative note, it may reduce the administration of medication to animals that require it. Of note, sow medications were also significantly lower in MR than in FC, which may support this latter consideration. However, if stockperson cautiousness was the reason for fewer medications in sows and particularly in piglets, then it would be expected that more piglets would have succumbed to the effects of poor health. This was not the case as there was no difference in liveborn mortality between the two treatments, and in fact, piglet removal for ill thrift was low in the MR sows. Future efforts should be made to quantify how stock people work in the two systems, and whether there are clinical or diagnostic shifts in sow and piglet health. Regardless, this finding implies that antibiotic use was lower in the MR than in the FC system, an encouraging observation given the increasing pressure towards the judicious use of antibiotics.

## 5 Conclusion

In conclusion, the number of piglets born and weaned was comparable between FC and MR treatments, and, given the identical space requirements, similar production outputs were achieved in both farrowing systems. Although there was a tendency for higher piglet mortality in MR, this was confined to the period immediately following farrowing and may be resolved by stabilising parity distribution or other management strategies. This is partially offset by the reduction in piglet medication and late fostering removal of ill-thrift piglets from MR sows. The uniquely comparable performance of the MR to the FC should question the sentiment that larger pen sizes result in improved piglet survival and provide a commercially viable, close-confinement-free option to replace the traditional farrowing crate.

## Data Availability

The raw data supporting the conclusions of this article will be made available by the authors, without undue reservation.
